# Virginia State Dental Association

**Published:** 1885-12

**Authors:** 


### Proceedings of Virginia State Dental Association.

SIXTEENTH ANNUAL SESSION.

The Association met in the City of Richmond, on Tuesday, October 6th, 1885, in the parlor of Ford's Hotel.

After the calling of the Association to order by the President, Dr. J. R. Woodley, of Norfolk, and the members present responding to their names, the minutes of the last session were read and approved.

The Executive Committee reported favorably upon the names of Drs. W. Y. Canada and H. W. Canada, of Halifax County; E. P. Bradley, Danville; W. L. Bacon, Lexington; E. S. Fawcett, Alexandria; Chas. T. Lindsay, Rockingham; F. A. Lee, Thos. E. Craddock, Ed. P. Parsons, Lynchburg, and they were elected to active membership.

Dr. Mercer, from the Committee on Dental Legislation reported progress, and hoped to get the bill through the next Legislature.

Appropriate hours were selected for the sessions of the Association, and also for clinics, of which the latter formed a most interesting portion of the exercises, and the skill of Dr. Gingrich, of Norfolk, Dr. J. Hall Moore and Dr. McCrown, of Richmond, was witnessed and admired by a large proportion of the members.

On motion of Dr. Moore, the Association re-affirmed its previous action in declining to authorize any of its officers to make appointments of beneficiary students in any dental college.

Dr. Gingrich, from the Committee on Operative Dentistry, presented a most able report, taking as his theme, "Lack of Operative Skill, some of the causes and a probable remedy. In a word, Failures and their prevention."

The subject was handled in a masterly manner, and received the commendation of all, especially of that able and fluent speaker, Dr. Thackston. The subject was thoroughly discussed, showing the earnest desire of all to aim to higher attainments, so as to agree with the report in rendering to all patients the very best service. Under this head "the use of cocaine" was discussed, but after an interchange of opinions it was concluded that the dentist's millenium had not yet come as was hoped when this salt was first introduced.

After this report was passed the regular order of business was suspended and the President delivered his annual address.

Dr. Thackston congratulated the Association in his usual felicitous way upon such appropriate remarks from the President, thinking that the lessons of the address were ample compensation for the sacrifices made in closing their offices and attending the sessions of the Association. He urged all, especially the younger members, to follow out the pathway which had been pointed out to them in the address.

Dr. Mercer, on behalf of the younger members, thanked the President for the lessons taught.

The Association then proceeded to ballot for officers for the ensuing year, which resulted in the election of the following:

President, Dr. W. H. Gingrich, Norfolk.

First Vice-President, E. P. Parsons, Lynchburg.

Second Vice-President, A. C. Smith, Louisa.

Third Vice-President, E. P. Bradley, Danville.

Treasurer, J. F. Thompson, Fredericksburg.

Recording Secretary, G. F. Kelsee, Richmond.

Corresponding Secretary, J. H. Moore, Richmond.

Who were duly installed into their respective offices.

Drs. Bacon, Craddock and Lee were elected as the Executive Committee.

Drs. Thackston and Thompson each presented able and interesting reports on the subject of Dental Education, which, upon discussion, showed that this subject was not threadbare.

Dr. Mercer, on behalf of the Richmond dentists, invited the Association to take a recess on the afternoon of the third day and accept of a drive to the various points of interest in and about the city, which was accepted with the cordial thanks of the Association.

The merits of the Southern Dental Association were presented in a communication from its President, Dr. Wardlaw, and discussed by the members, and Drs. Mahony, Moore, Woodly,

Parsons, Beadles, Bacon and Faucett were appointed as representatives to the next session of the Association, with request to invite that Association to hold its session of 1887 in Virginia.

Letters were read from Professors Taft and Hodgkin, and Doctors Waters, Hunt and Caulk, corresponding members regretting their inability to be present.

On motion, the Recording Secretary was instructed to furnish Dr Taft, Editor of the DENTAL REGISTER, an abstract of the proceedings for insertion in his journal.

A vote of thanks was returned to the railroads, daily press, and Ford's Hotel, for courtesies extended.

No further business appearing, the Association adjourned to meet at the Natural Bridge on the second Wednesday in August, 1886.

